# Methods for evaluating technical innovations in the implementation of energy-saving measures in enterprises

**DOI:** 10.1016/j.mex.2022.101658

**Published:** 2022-03-04

**Authors:** Svetlana Drobyazko, Tetiana Hilorme

**Affiliations:** a The European Academy of Sciences Ltd, 71-75 Shelton Street Covent Garden, London WC2H 9JQ, UK; b Oles Honchar Dnipro National University, Gagarina 78, Dnipro, Ukraine

**Keywords:** Primary data collection, Project, Energy saving, Network schedule, Specifications

## Abstract

The method of calculating the cost of implementing the system of drying of fuel by gases for reduction of expenses of fuel for the TPP-210A boiler unit is proposed. This method allows to determine changes in the technical parameters of the four options for the use of fuel for the unit: normal fuel, without drying, with drying in a closed system, and with drying in an open system (the last 3 options involve degraded fuel). Processed fuel requirement will decrease by 14 %. Project economic efficiency is 0.3 US dollars/US dollars. The project payback period is 3.33 years. Program (Project) Evaluation and Review Technique (PERT) was used as the data collection methodology. The network schedule of the planning process and determination of its design parameters of the energy-saving project development are built. As an additional method for the collection of primary data to calculate the cost, the primary data collection tool ‘1C Enterprise’ software product was used.

• To determine the cost of the project, the methodology for estimating the implementation of the project of the gas fuel drying system based on the determination of capital costs and technological capabilities of technical equipment is improved;

• It is proposed to use the method of network planning and management in the system of development of energy-saving projects as it allows to determine the design parameters to increase the efficiency of research activities;

• This methodology for estimating the technical and economic calculation of the implementation of the project of the gas fuel drying system for cost reduction may be of interest to industrial enterprises and thermal power plants that use steam boilers.

## Graphical abstract

**Figure:**
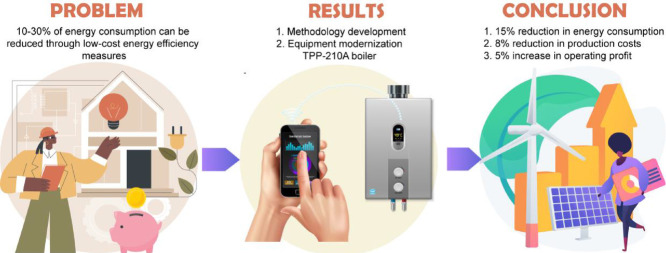

## Direct submission

Specifications Table

**Table:** 

Subject:	Energy
More specific subject area:	Energy-saving technologies at industrial enterprises
Method name:	Methods for evaluation of technical innovations in the implementation of energy-saving measures in enterprises
Name and reference of original method:	S. Drobyazko, T. Hilorme, V. Shevchenko, O. Yudina, Assessment of energy sustainability and development of companies based on cognitive modelling, E3S Web Conf., 277 (2021) 02006. doi.org/10.1051/e3sconf/202127702006
Resource availability:	NA

## Method details

### Methods for evaluation of energy-saving projects

When implementing energy-saving measures, the appropriate methodology is considered, which has the following sections [1]: preamble (summary, goals (objectives)); technical and economic characteristics of the basic energy scheme; measures to improve the management of this energy scheme; calculation of capital investments for technical re-equipment of the projected energy scheme; calculation of savings in current energy system costs; calculation of the energy system operating costs; calculation of prime cost reduction; determining the effectiveness of the suggested measures; and calculation of savings from the reduction of payments for the environmental intervention of an enterprise. The preamble provides a brief description of the problems to be solved when designing the energy scheme of energy-saving measures, the scheme of technical and economic calculations, and defines the expected results to be obtained after the implementation of organizational and technical measures of the project. The technical and economic characteristics of the basic energy scheme provide a general description of the technological object and the energy system serving it. The scheme of the organizational structure of the economic entity is defined in conjunction with the energy system.

Additionally, it is necessary to provide technical (capacity, consumption, costs, losses, etc.) and economic (book value) characteristics of the energy system; calculation of the cost of energy produced (consumed) by the energy system; effective operating time; staff salaries; the volume of output; calculation of the prime cost of products; prices for products and energy resources; and indicators that characterize the operation of this energy system [2]. Measures to improve the management of the energy scheme. Based on the results of the analysis of the operation of the energy system, identification of shortcomings in its work, as well as information obtained in the process of scientific research suggestions are made regarding improvement of the existing energy system for energy saving.

**Technical and economic calculation of the implementation of the project of the system of fuel drying with gases for reduction of fuel consumption**

To increase fuel use efficiency and the efficiency of the boiler, it is suggested to install a system of fuel drying with exhaust gases of the technological process [3]. These changes will improve the operation of basic process equipment and fuel specifications. The specifications of the TPP-210A boiler unit after modernization are given in Table 1.

**Table 1:** Specifications of the TPP-210A boiler unit after modernization.

Indicator			Options Fuel of degraded quality		
	Measurement units	Normal fuel	Without drying	With closed-circuit drying	With open-circuit drying
		1	2	3	4
Fuel calorific value	KJ/kg	27,445	20,950	20,950	22 877
Fuel ash content	%	15.2	27.9	27.9	30.6
Fuel humidity	%	5	8.9	8.9	0.5
Steam rate	t/h	375	375	375	375
Superheated steam pressure	МPа	24	24	24	24
Superheated steam temperature	^о^С	565	565	565	565
Secondary steam rate	t/h	300	300	300	300
Secondary steam pressure	МPа	3.5	3.5	3.5	3.5
Secondary steam temperature	^о^С	570	570	570	570
Heat loss with exhaust gases	%	4.7	7.48	7.77	5.92
Heat loss with mechanical underburning	%	2.0	2.5	2.5	2.436
Heat loss to the environment	%	0.2	0.53	0.53	0.53
Boiler COP	%	92.66	89.13	88.83	90.74
Boiler useful heat	GJ/y	1062	970.8	980.04	957.8
Estimated fuel consumption	t/y	54.67	67.63	60.67	60.67

Calculation of capital investments for technical re-equipment of the designed energy system. Capital investments in re-equipment of the designed energy system consist of the cost of energy equipment and energy transmission means (power lines, pipelines, etc.).

The cost of buildings (if provided in the project) required to accommodate the central control panel of the energy system is calculated according to the volume of the design size of the building and the average cost per 1m3 of the building according to rules and regulations or the actual cost of construction [4].

The cost of energy equipment, computers, and so on is determined based on the specifications according to the current price lists. The costs of transportation, installation, commissioning, spare parts, and other costs are also considered. In addition, it is necessary to consider the cost of developing design documentation [5].

`

After the introduction of the fuel drying system using process exhaust gases, the output in volume terms will not change. However, the need for process fuel will decrease by 14 %. Consequently, the prime cost of production will decrease.

To install burners of the selected type, it is necessary to dismantle the burners that were installed earlier. The total amount of capital investments for technical re-equipment of the projected energy system can be calculated as per Eq. 1:

(1) $$K_{\text{total}}^{e}=K_{1}+K_{3}{+}_{3}+K_{4}+K_{5}+K_{6}-K_{7}$$

where К_1_ is capital expenditures for the construction of buildings, dollars; К_2_ is capital expenditures for power equipment, dollars; К_3_ is capital costs for installation work (accepted according to actual data or 15–20 % of К_1_ + К_2_), dollars; К_4_ is overhead (transport, preservation, spare parts, etc.) accepted at increased rates (5 %) of the cost of equipment, dollars; К_5_ is capital costs for the development of design and engineering documentation, dollars; К_6_ is other costs (cost of materials, devices, equipment, which are not part of the cost estimate and other costs), dollars; and К_7_ is the residual value of the dismantled equipment, dollars.

When it is not planned to build a new building, the general cost estimate for power equipment is given in Table 2.

**Table 2:** Cost estimate for power equipment.

Item no.	Name of works and costs	Measurement units	Number of measurement units	Estimate cost, US dollars	
				Units	Total cost
1	Control unit	pcs	1	63,850	63,850
2	Computer	pcs	1	12,770	12,770
3	Power supply lines	US dollars	-	-	86,187
4	Installation work	US dollars	-	-	33,150
5	Capital costs for the development of design and engineering documentation	US dollars	-	-	5700
	Total costs	-	-	-	201,657

Source: Research calculations.

Thus, the total cost of capital investments for the project implementation is 201,657.00 US dollars.

The calculation of savings in current costs of the power system is determined according to the cost structure.

The calculation of the change in costs related to the use of raw materials is determined for each type separately by Eq. 2:

(2) $$E_{st}=\left(N_{1}-N_{2}\right)\times P_{st}\times Q$$

where $E_{st}$ is the annual cost savings for raw materials or supplies, US dollars; $N_{1},N_{2}$ is the rate of consumption of raw materials or materials per unit of production before and after the organizational and technical measures, respectively, natural units; $P_{st}$ is the price of raw materials or supplies, US dollars/natural units; and Q is production output during the year after the introduction of organizational and technical measures, natural units.

Changes in the costs related to the use of raw materials and supplies are not provided for. Calculation of changes in costs related to the use of fuel, steam, gas, water and electricity. Cost savings related to fuel use are determined by Eq. 3:

(3) $$E_{fuel}=\left(N_{1}-N_{2}\right)\times P_{fuel}\times T_{ef}$$

where *Е*_fuel_ is annual fuel savings, US dollars; *N_1_* and *N_2_* are fuel consumption rate per unit time, before and after organizational and technical measures, respectively, natural units/hour; *P_fuel_* is fuel price, US dollars/natural units; and T_ef_ is effective operating time of the equipment during the year, hours.

The effective worktime fund of the equipment is determined by Eq. 4:

(4) $$T_{ef}=T_{1}\times n_{1}\times T_{2}-T_{3}-T_{4}$$

where T_1_ is the duration of the operating shift, hours; $n_{1}$ is number of operating shifts, units; $T_{2}$ is calendar time fund, hours; T_3_ is duration of planned and preventive repairs of equipment, hours; and T_4_ is duration of equipment downtime, hours.

The mode of operation of the basic enterprise is continuous in three shifts lasting 8 hours, the effective worktime fund of equipment is defined by Eq. 4. After the project implementation, the fuel (thermal coal) consumption will be reduced from 1283,213 t/hour to 1146,946 t/hour. Calculation of operating costs for the energy system. Additional costs related to the operation of the energy system consist of additional depreciation deductions (*В_а_*). The calculation of the depreciation amount also considers the additional capital costs related to the implementation of measures. Thus, the total annual amount of additional costs *∆В* is obtained in Eq. 5:

(5) $$\Delta B=B_{a}$$

Depreciation costs (*В_а_*) are calculated based on data on the average annual value of fixed assets (FA) of a certain group (F_і_) and the depreciation rate established for this group (*Н_аі_*) using Eq. 6:

(6) $$B_{a_{i}}=F_{i}\times H_{a_{i}}$$

where *F_і_* is the average annual value of fixed assets of a certain group, US dollars; Н_аі_ is depreciation rate of the corresponding *i*-th group of fixed assets of an enterprise, percentage; and *і*-th group of fixed assets of an enterprise.

The depreciation rate is determined by the straight-line method using Eq. 7:

(7) $$H_{a}=\frac{1}{T_{oper}}$$

where T_oper_ is the useful life (operation) of fixed assets in years.

The annual amount of depreciation (A) is equal to the amount of depreciation of all groups of fixed assets according to Eq. 8:

(8) $$B=\sum B_{a_{i}}$$

According to Table 2, the expenses for power equipment (group 4 of fixed assets) is equal to 201,657 US dollars. The calculation of depreciation deductions is given in Table 3. The calculation of the depreciation amount also considers the additional capital costs related to the implementation of measures.

**Table 3:** Calculation of the amount of depreciation deductions for the project.

Group of fixed assets	Average annual value of fixed assets (Fi), US dollars	Useful life (operation) of fixed assets (*Т_oper_*), years	Depreciation rate (*Н_аі_*), percentage (Eq. 7)	Annual depreciation (*В_а_*), US dollars (Eq. 8)
Group 3 (buildings, structures, transmitting devices)	–	15	–	–
Group 4 (machinery and equipment)	201,657	5	–	40,331.4
Group 5 (transportation means)	–	5	–	–
Group 6 (tools, inventory)	–	4	–	–
Sum	201,657	Х	Х	40,331.4

Source: Research calculations.

Thus, the total annual amount of additional costs ∆В is obtained:

∆В = 40,3N31.4 US dollars/year

Economic efficiency is determined by the ratio of the result (effect) to costs. The result is the profit, which is determined as follows: R&D profit = 26,787 US dollars – 20,605 US dollars = 6,181 US dollars.

Then the project economic efficiency = result/costs = 6,181/20,605 = 0.3 US dollars/US dollars.

The project payback period is determined as follows: 20,605/6,181= 3.33 years.

As noted above, the sufficiency of the payback period is determined by the investor.

Determining the parameters of the organization of research work. The organization of research work has the following elements: patent search; analytical literature review; and experimental part, which includes determining the optimal conditions for the experiment [6]. For rational use of time during research work, the method of network planning and management was used, which is based on the estimation of parameters of the network schedule. By estimating the full paths on the graph, the total duration of research work is determined. For this purpose, the scheduled plan of research work given in Table 4 is used.

**Table 4:** Research work plan.

Work name	Work code	Work duration t_ij_, days
Formulation of the problem	0-1	1
Drawing up an experiment plan	1-2	2
Literature review	2-3	20
Substance preparation	3-4	2
Preparation of utensils and equipment	3-6	1
Examination of samples of ARPD	4-7	2
Preparation of solvent compositions	6-7	2
Solubility study by static method	6-8	3
Solubility study by dynamic method	7-8	3
Density measurement	7-9	4
Graph plotting	9-10	4
Verification by the head of research work	10-11	5
Execution of ‘Labor protection’ section	10-12	3
Design of the experimental part	11-14	7
Execution of ‘Organizational and economic part’ section	12-13	3
Design of the research paper	13-14	7
Review and defence	14-15	2
Total (total duration of all works and expectations), days		71
Same but in %		100

**Figure 1:**
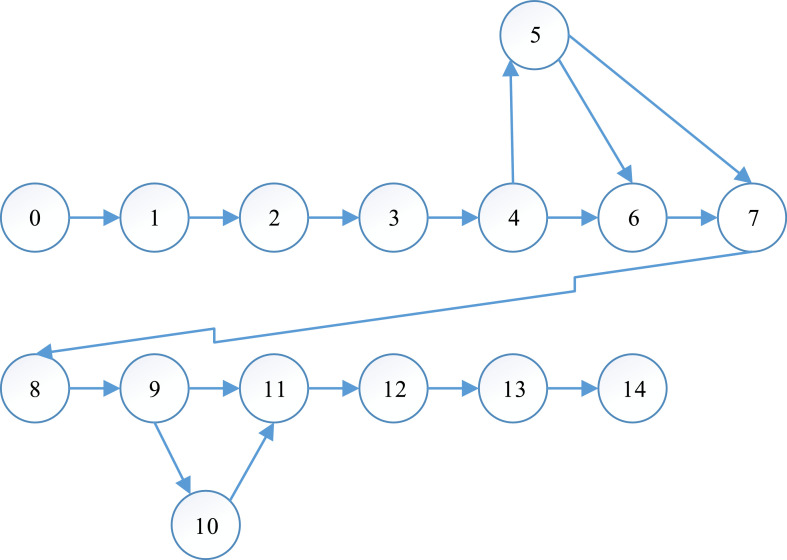
Network schedule of research work.

Source: Research calculations.

Based on the listed works and sequence of their execution, the network schedule (Fig. 1) is built based on which the critical path is determined using tabular and graphic methods. This is the path that has maximum effectiveness.

The calculation of the duration of all possible paths is given in Table 5.

**Table 5:** Calculation of path duration in the organization of research work.

Item no.	Path no.	Path work scope	Path work duration, days	Path duration, days
1	L_1_	0, 1, 2, 3, 4, 6, 7, 8, 9, 11, 12, 13, 14	1, 2, 20, 2, 2, 3, 4, 4, 3, 3, 16, 2	62
2	L_2_	0, 1, 2, 4, 6, 7, 8, 9, 10, 13, 14	1, 2, 20, 2, 2, 3, 4, 4, 5, 7, 2	52
3	L_3_	0, 1, 2, 3, 5, 6, 7, 8, 9, 11, 12, 13, 14	1, 2, 20, 1, 2, 3, 4, 4, 3, 3, 16, 2	61
4	L_4_	0, 1, 2, 3, 5, 6, 7, 8, 9, 10, 13, 14	1, 2, 20, 1, 2, 3, 4, 4, 5, 7, 2	51
5	L_5_	0, 1, 2, 3, 5, 7, 8, 9, 11, 12, 13, 14	1, 2, 20, 1, 3, 4, 4, 3, 3, 16, 2	59
6	L_6_	0, 1, 2, 3, 5, 7, 8, 9, 10, 13, 14	1, 2, 20, 1, 3, 4, 4, 5, 7, 2	49

Source: Research calculations.

L_1_ is the critical path. Therefore, the duration of the critical path of research work is 62 days. We determine the parameters of the network schedule using Eq. 9:

(9) $${t_{ij}}^{early\;start}={t_{i}}^{early\;date}$$

where t_ij_^early start^ is characterized by the duration of the critical path from the initial event to the start of this work; t_i_^early date^ is the early date of the *і-*th event determined by the duration of the critical path from the initial event to the *і-*th event. It takes into account that the initial event takes place at zero time.

This means that the early start of the *і-*th event is equal to the early start of the work *іj* (Table 5). The early end of the work is determined by the duration of the critical path from the initial event to the end of the work using Eq. 10:

(10) $${t_{ij}}^{early\;end}={t_{i}}^{early\;date}+t_{ij}$$

where t_ij_^early end^ means early end; t_i_^early date^ is the late date of the *j-*th event. This is a period that will not delay the implementation of the final event or disrupt the general deadlines.

The indicator of late date of the j-th event is calculated using Eq. 11:

(11) $${t_{j}}^{late\;start}=L_{critical\;path}-t\left[L{\left(jc\right)}_{max}\right]$$

where t_j_^late start^ is the late start of work. This is a period that will not delay the implementation of the final event or disrupt the general deadlines.

The calculation of the late start of work is based on Eq. 12:

(12) $${t_{ij}}^{late\;start}={t_{i}}^{late\;date}-t_{ij}$$

where t_ij_^late end^ is late end of work (t_ij_^late end^ = t_i_^late date^ is the latest allowable end of work); Р_і_ is the time reserve, which is the difference between t_i_^late date^ and t_i_^early date^. There are full and free time reserves.

Full reserve of work time (Р_ij_^full time^), which shows how much it is possible to increase the duration of work, so that the maximum full path that passes through it, not exceeding the duration of the critical path (Eq. 13):

(13) $$P_{ij}^{latedate}=t_{j}^{latedate}-t_{i}^{earlydate}-t_{ij}$$

where Р_ij_^free time^ is a free reserve of work time showing that part of the full reserve, which can be used only for this work, without changing the early dates of subsequent works (Eq. 14):

(14) $$P_{ij}^{freetime}=t_{j}^{earlydate}-t_{i}^{earlydate}-t_{ij}$$

The calculation of network schedule parameters is given in Table 6**.**

**Table 6:** Time parameters of the network schedule of research work, days.

No.	i - j	t_i-j_	t_i-j_^early start^	t_i-j_^early end^	t_i-j_^late start^	t_i-j_^late end^	Р_ij_^full time^	Р_ij_^free time^
1	0-1	1	0	1	0	1	0	0
2	1-2	2	1	3	1	3	0	0
3	2-3	20	3	23	3	23	0	0
4	3-4	2	23	25	23	25	0	0
5	3-6	1	23	24	24	25	1	1
6	4-7	+ 2	25	27	25	27	0	0
7	6-7	2	24	26	25	27	1	0
8	6-8	3	24	27	27	30	3	2
9	7-8	3	27	30	27	30	0	0
10	7-9	4	30	34	30	34	0	0
11	9-10	4	34	38	34	38	0	0
12	10-11	5	38	43	48	53	10	10
13	10-12	3	38	41	38	41	0	0
14	11-14	7	43	50	53	60	0	0
15	12-13	3	41	44	41	44	0	0
16	13-14	+ 16	44	60	44	60	0	0
17	14-15	2	60	62	60	62	0	0

The calculation of the results of the early and late start of events, as well as time reserves, is given in Table 7.

**Table 7:** Results of early and late start of events, as well as time reserves.

t_i_	0	1	2	3	4	5	6	7	8	9	10	11	12	13	14
t_i_^early date^	0	1	3	23	25	24	27	30	34	38	43	41	44	60	62
t_i_^late date^	0	1	3	23	25	25	27	30	34	38	53	41	44	60	62
Р_і_	0	0	0	0	0	1	0	0	0	0	10	0	0	0	0

After planning the period of research work, it is necessary to calculate the cost estimate for the study. The cost estimate for the study includes the following [6]: costs of raw materials and supplies; the costs of wages of staff engaged in research and social security contributions; energy costs; equipment depreciation costs; and overhead costs.

It is necessary to determine the economic constraints of the calculations. Thus, tax legislation changes affect the definition of depreciation methods and groups of fixed assets; taxation of wages and other expenses. If a preferential regime for energy-saving projects as an energy security measure is in place in a country, it is necessary to consider tax benefits and preferential regimes. Additionally, the presence of a minimum wage in the country is another limiting factor. If the level of the minimum wage increases, the expenses for project payments grow accordingly thereby increasing the total prime cost. Apart from direct factors, indirect factors also, particularly changes in the inflation index, also influence the prime cost.

## Experimental design, materials, and methods

The effectiveness of research work is determined by the correctly chosen area of work and economic costs. When performing research work to increase its effectiveness, it is necessary to properly plan and manage the process. Among modern methods of scientific planning and management, the method of network planning and management (NPM) has become the most widespread [7]. One of the main steps in using the method of NPM is to build a network model — a schedule of the planning process — and determine its design parameters. An example of the organization of joint work of agents is ‘IBM Lotus Notes’ of ‘IBM Lotus Domino’, which can plan and work with e-mail and is analogous to ‘Outlook’. ‘IBM Lotus Sametime’ supports web conferencing and mobile networking. ‘IBM Lotus Quicker’ is designed for joint work of staff inside and outside the enterprise, and ‘Lotus Connections’ enables people to work together on joint projects.

## Conclusion remarks

The organization of a study has the following elements: patent search; analytical literature review; and experimental part, which includes determining the optimal conditions for the experiment.

For the rational use of time during research work, the method of network planning and management was used, based on the estimation of parameters of the network schedule. According to the assessment of complete paths, the graph determines the total duration of research work.

One of the limitation of the proposed study is based on the principle of Ceteris paribus. This means that it is necessary that the economic interests of alpha-stakeholders in the implementation of alternative projects for indoor climate systems are the same. Alpha stakeholders form their requirements per the goals and motivations and influence the project based on their interests, professional competencies and degree of involvement in its implementation.

The implementation of key requirements based on a basic approach can be guaranteed by combining traditional development with the creation of new attributes of its key element, the energy system. It is suggested to consider the expected effects of the project depending on the group of stakeholders: energy companies, end consumers, regulators and the state and society as a whole. It is necessary to note the following specifics of the requirements of the considered groups of stakeholders.

Firstly, group 2 (‘State’) and group 3 (‘Consumers’), in addition to the requirements for the state of the energy system of the country, have the corresponding requirements/expected effects for group 1 (‘Energy companies’). This is because energy companies provide energy services to other groups of stakeholders.

Secondly, group 1 (‘Energy Companies’) and group 2 (‘State’) have the following common requirements/expected effects: reduction of electricity losses and improvement of energy system management processes. It is the reduction of electricity losses that forms the expected profits of energy companies, and for the state, this parameter makes it possible to build an energy-efficient society. Improvement of energy system management processes satisfies the condition for the development of power systems from the standpoint of these groups of stakeholders.

Thirdly, the requirement regarding the ‘possibility to sell electricity on the market’ applies only to the population as a subgroup of end consumers.

To offset possible limitations and violations of the balance of interests, competencies and the degree of involvement of project agents, it is important to note some recommendations for its implementation [8]:

1) The built alternative option allows making decisions on reducing the use of natural gas. This is possible due to: the introduction of the latest energy-saving technologies used to reduce the energy consumption of production and increase the competitiveness of products; carrying out engineering and technical, scientifically implemented measures.
2) Under the current conditions of world economic development, industrial enterprises implement an optimal strategy based on energy saving and consider energy efficiency as an important component of innovative industrial development.
3) Volumes and the range of applications of renewable energy sources, which will partially replace natural gas, are growing.

## Declaration of Competing Interest

The authors declare no conflict of interest.

## Appendix Supplementary materials

Image, application 1

## References

[1] Drobyazko S., Wijaya S., Blecharz P., Bogachov S., Pinskaya M. (2021). Modeling of Prospects for the Development of. Regional Renewable Energy. Energies.

[2] Drobyazko S., Skrypnyk M., Radionova N., Hryhorevska O., Matiukha M. (2021). Enterprise energy supply system design management based on renewable energy sources. Global J. Environ. Sci. Manage.

[3] Drobyazko S., Hilorme T., Solokha D., Bieliakova O. (2020). Strategic policy of companies in the area of social responsibility: Covid-19 challenges. E3S Web Conf.

[4] Drobyazko S. (2020). Proceedings of the 35th International Business Information Management Association Conference (IBIMA). Vision 2025: Education Excellence and Management of Innovations through Sustainable Economic Competitive Advantage.

[5] Hilorme T., Tkach K., Dorenskyi O., Katerna O., Durmanov A. (2020). Decision making model of introducing energy-saving technologies based on the analytic hierarchy process. Journal of Management Information and Decision Sciences.

[6] S. Drobyazko, T. Hilorme, V. Shevchenko, O. Yudina, Assessment of energy sustainability and development of companies based on cognitive modeling, E3S Web Conf., 277 (2021) 02006. doi.org/10.1051/e3sconf/202127702006

[7] Hilorme T., Nazarenko I., Okulicz-Kozaryn W., Getman O, Drobyazko S. (2018). Innovative model of economic behavior of agents in the sphere of energy conservation. Academy of Entrepreneurship Journal.

[8] Nakashydze L., Hilorme T., Nakashydze I. (2020). Substantiating the criteria of choosing project solutions for climate control systems based on renewable energy sources. Eastern-European Journal of Enterprise Technologies.

