# Supplementary material for: Methods for evaluating technical innovations in the implementation of energy-saving measures in enterprises
Source: MethodsX. 2022 Mar 4;9:101658. doi: 10.1016/j.mex.2022.101658 (PMC8924305; doi:10.1016/j.mex.2022.101658)
Supplement: Supplementary file 1 [file mmc1.pdf]

ELSEVIER

Certificate of Elsevier Language Editing Services

The following article was edited by Elsevier Language Editing Services:

"Methods for evaluation of technical innovations in the implementation of energy saving measures in enterprises"

Authored by:

Svetlana Drobyazko

Date: 16-Feb-2022

Serial number: LE-232799-79396BBC8579
